# The burden of diabetic emergencies on the resuscitation area of a district-level public hospital in Cape Town

**DOI:** 10.1016/j.afjem.2021.05.004

**Published:** 2021-10-14

**Authors:** N. Lotter, S. Lahri, D.J. van Hoving

**Affiliations:** Division of Emergency Medicine, Stellenbosch University, South Africa

**Keywords:** Diabetes, Emergency, South Africa, Diabetic ketoacidosis, Burden

## Abstract

**Introduction:**

Diabetes and its complications continue to cause a daunting and growing concern on resource-limited environments. There is a paucity of data relating to the care of diabetic emergencies in the emergency centres of entry-level hospitals in Africa. The aim of this study was to describe the burden of diabetic emergencies presenting to the emergency centre of an urban district-level hospital in Cape Town, South Africa.

**Methods:**

The Khayelitsha Hospital Emergency Centre database was retrospectively analysed for patients presenting with a diabetic emergency within a 24-week randomly selected period. The database was supplemented by a retrospective chart review to include additional variables for participants with diabetic ketoacidosis (DKA), uncomplicated hyperglycaemia, severe hypoglycaemia and hyperosmolar hyperglycaemic state (HHS). Summary statistics are presented of all variables.

**Results:**

The prevalence of all diabetic emergencies was 8.1% (197/2424) (DKA n = 96, 48.7%; uncomplicated hyperglycaemia n = 45, 22.8%; severe hypoglycaemia n = 44, 22.3%; HHS n = 12, 6%). The median age was 48 years, with those presenting with DKA being substantially younger (36 years). A likely precipitant was identified in 175 (88%) patients; infection was the most common precipitant (n = 79, 40.1%). Acute kidney injury occurred in 80 (40.6%) cases. The median length of stay in the resuscitation area was 13 h (IQR 7.2–24) and 101 (51.3%) participants represented with a diabetic- related emergency within six months of the study period. The overall mortality rate was 5% (n = 10).

**Conclusion:**

This study highlights the high burden of diabetic emergencies on the provision of acute care at a district-level hospital. The high prevalence of diabetic emergencies (8%) consisted of DKA (48.7%), uncomplicated hyperglycaemia (22.8%), severe hypoglycaemia (22.3%), and HHS (6%). The high infection rate (40%) and the high percentage of patients returning with a diabetic emergency (51%) could be indicative of the need for improved community-based diabetic programmes.

## African relevance

● Non-communicable diseases like diabetes are on the increase, resulting in a high prevalence of diabetic emergencies presenting to emergency centres.
● Infection is the most frequent precipitating factor in diabetic emergencies.
● Half of patients re-presented with a diabetic-related emergency within six months.

## Introduction

Diabetes is a significant contributor to morbidity and mortality and remains a global health concern. An estimated 382 million people had diabetes in 2013, and this number is expected to double by 2035; the majority of this increase is expected in low-and middle-income countries [1]. The World Health Organization further projects that diabetes will be the seventh leading cause of death in 2030 [2]. In South Africa, an estimated 7% of economically active citizens have diabetes, and along with other non- communicable diseases, pose a major socio-economic threat to South Africa 3., 4..

Diabetes is globally one of the most prevalent chronic diseases amongst emergency centre patients 5., 6., and patients often present with a diabetic emergency (diabetic ketoacidosis (DKA), hyperosmolar hyperglycaemic state (HHS), uncomplicated hyperglycaemia and severe hypoglycaemia). Patients with diabetic emergencies should ideally be treated in intensive care or high care settings [7], but this may not always be possible [8]. As a result, these acutely ill patients are often treated for extended periods in the emergency centre [9]. This places undue strain on emergency centre staff and resources, as these patients require precise monitoring and ongoing management [7].

There is currently a paucity of data about the burden of diabetic emergencies on emergency centres at entry-level hospitals, as most studies focussed on intensive or high care units within well-resourced countries. South Africa has a mounting burden of diabetes and other non-communicable diseases [10], and knowledge of the burden of diabetic emergencies within the local context is essential as part of the effort to reduce diabetic-related morbidity and mortality. The study set out to describe the burden of diabetic emergencies on the emergency centre of a district-level hospital in Cape Town, South Africa.

## Methods

A retrospective analysis of a prospectively collected observational database was conducted at Khayelitsha Hospital covering a period of 24 randomly selected weeks between 1 January 2017 and 30 June 2018. The database was supplemented by a retrospective chart review to include additional variables. The study was approved by the Health Research Ethics Committee of Stellenbosch University (S18/10/215).

Khayelitsha Hospital is a 340-bed hospital situated in the partially informal township of Khayelitsha, Cape Town. It serves a health district with a population just fewer than 500,000 people; a substantial proportion of which are unemployed (38%) [11]. The resuscitation area within the emergency centre consists of four adult-sized beds and one paediatric/neonatal resuscitation cot. Each bed is individually equipped with non-invasive and invasive monitoring tools, a fully stocked resuscitation trolley, a ventilator, and a defibrillator. A blood gas machine is also situated within the resuscitation area. Patients managed within the resuscitation area are selected based on a high acuity level on the South African Triage Scale or by a senior physician's clinical gestalt [12]. Other than theatre, the resuscitation area is the only place capable of continuous patient monitoring, as the hospital does not have a high care or intensive care unit.

The Khayelitsha Hospital Emergency Centre database is a prospectively collected observational database and has previously been described [6]. In brief, data is captured electronically, coded and stored onto a password-protected server. A decoding sheet is separately stored. The database has been registered at the Stellenbosch University Health Research Ethics Committee (Ref: N15/10/107).

Convenience sampling was used, and 24 weeks within 18 months was randomly selected (using a computer randomizer). The sample size was limited due to restricted resources; however, it was expected to be representative of the population. All patients who presented to the resuscitation area with a diabetic emergency (DKA, HHS, uncomplicated hyperglycaemia or hypoglycaemia) were eligible. Patients presenting with uncomplicated hyperglycaemia were included as many patients with elevated glucose levels are unable to produce urine at triage. These patients are often severely ill despite looking well, and true hyperglycaemic emergencies (DKA, HHS) have previously been missed as access to serum ketones (laboratory or point-of-care) is limited. All patients with elevated or decreased glucose levels are thus managed within the resuscitation area until a senior clinician have evaluated the patient. Patients who presented with a diabetic emergency in the absence of pre-existing or newly diagnosed diabetes were excluded (e.g. hyperglycaemic stress response); this was mainly done by clinical gestalt, as HBA1C is not routinely ordered. Patients with missing folder numbers or medical notes were also excluded.

Diabetic emergencies were defined using criteria from the American Diabetes Association (ADA) and the Society for Endocrinology, Metabolism and Diabetes of South Africa (SEMDSA) 7., 13.. DKA was defined as hyperglycaemia with glucose >13.9 mmol/l, metabolic acidosis with pH <7.3 and bicarbonate <18 mmol/l, and presence of ketonemia (>3 mmol/l). The presence of urine ketones was used as a surrogate marker when indicated, as point-of-care serum ketones were not readily available in the emergency centre and laboratory requested serum ketones are often associated with a delay in obtaining results. The serum pH was used to classify severity: mild (7.25–7.3), moderate (7.0–7.24), severe (<7.0). Resolution of DKA was determined by an improvement of acidosis (pH >7.3, bicarbonate >18 mmol/l) and resolution of ketonuria. HHS was defined as severe hyperglycaemia (serum glucose >33.3 mmol/L), hyperosmolality (serum osmolality >320 mOsm/kg), marked dehydration and the absence of significant acidosis (pH > 7.3, bicarbonate >15 mEq/L); ketonuria may be slight or absent. Uncomplicated hyperglycaemia was defined as random blood glucose >11.1 mmol/l in the absence of ketonuria and acidosis. Hypoglycaemia was defined as a blood glucose level <3.9 mmol/l with an altered level of consciousness.

The Triage Early Warning Score (TEWS) was used to determine patient acuity. The TEWS is a composite score of physiologic parameters measured at arrival to the hospital. It forms part of the South African Triage Scale and categorizes patients as non-urgent (green), urgent (yellow), very urgent (orange), and emergency (red) [12].

A single trained data collector (NL), not blinded to the study's objective, was responsible for the abstraction of information. Data were not double checked due to restricted resources. Data was collected after a decoded cleaned extract of the electronic database has been obtained (cleaned: copied into an Excel spreadsheet with all non-diabetic emergencies removed). Duplicated or conflicting data entries were identified by evaluating time of entry on the electronic database and electronic hospital records. Inclusion or exclusion of data entries were determined by consensus after discussing with a second reviewer (SL).

Data was collected after a decoded cleaned extract of the electronic database has been obtained (cleaned: copied into an Excel spreadsheet with all non-diabetic emergencies removed). The password-protected Excel spreadsheet was further populated using the hospital's electronic clinical records. Data collected included diagnosis, demographic profile, clinical presentation, precipitating factors, comorbidities, biochemical profile, diagnostic tests performed, interventions received while in the resuscitation area, length of stay in the resuscitation area, length of hospital stay, disposition from resuscitation area, and in-hospital mortality. Patient folder numbers, the primary identifier used in the database was removed once the entire data collection was complete. A pilot study was conducted on ten participants to standardise data abstraction (data was included).

Incomplete data points were excluded from the analysis. Summary statistics were used to describe all variables. Categorical data were summarised using frequency counts or percentages, and distributions of variables were presented as two-way tables. Medians and means were used as the measures of central tendency for ordinal and continuous responses and standard deviations or quartiles as indicators of spread. Analysis was performed using Microsoft® Excel for MAC Version 16.30 (19101301).

## Results

**Fig. 1:**
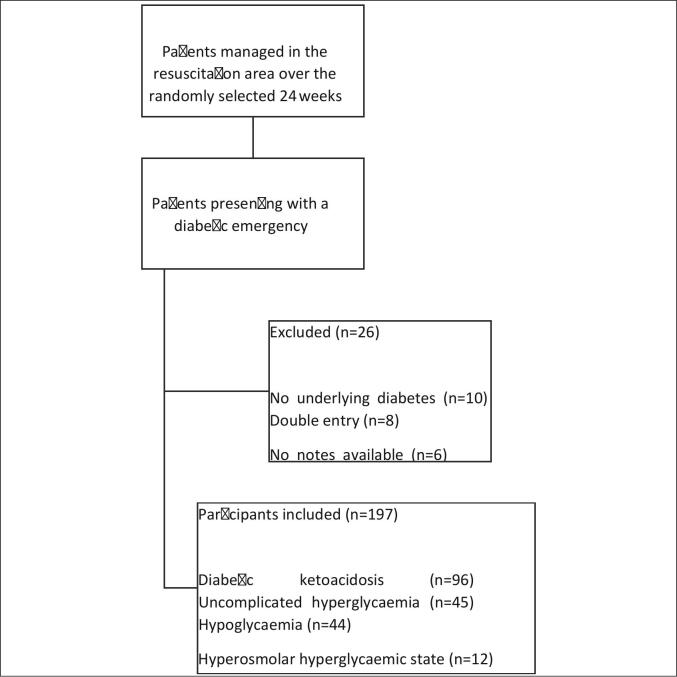
Flow diagram of study participants.

A total of 197 patients with a diabetic emergency were included after 26 cases were excluded. The prevalence of all diabetic emergencies was 8.1% (197/2424), with DKA occurring most frequently (48.7%, 96/197) (Fig. 1).

Demographic details of participants are given in Table 1. Most participants were female (n = 113, 57.4%). The median (25th–75th percentile) age was 48 (31–62) years, with participants diagnosed with DKA being substantially younger (36 (25–46) years). The majority of the participants (n = 135; 68.5%) had type 2 diabetes mellitus (T2DM), and overall, 31 (15.7%) were newly diagnosed with diabetes. A total of 135 (68.5%) participants presented outside regular office hours (weekdays 08 h00-15 h59) and 101 (51.3%) re-presented with a diabetic-related emergency within six months; with 60 (59.4%) re-presenting within 30 days. The median (25th–75th percentile) age for re- presenters in this study was 48 (29–61) years. Most were T2DM (n = 70; 69.3%), 66 (65.3%) on insulin, and 32 (31.7%) on oral anti-diabetic agents. The in-hospital mortality rate amongst the re-presenters was 6.9%.

**Table 1:** Demographic details of patients admitted with a diabetic emergency to the resuscitation area of Khayelitsha Hospital.

	DKA (n = 96) n (%)	HHS (n = 12) n (%)	Uncomplicated hyperglycaemia (n = 45) n (%)	Severe hypoglycaemia (n = 44) n (%)	Overall (n = 197) n (%)
Male	48 (50%)	7 (58.3%)	17 (37.8%)	12 (27.3%)	84 (42.6%)
Median age (years) (Q1-Q3)^a^	36 (25–46)	63 (56–71)	54 (44–63)	62 (54–70)	48 (31–62)
New diabetic diagnosis	19 (19.8%)	4 (33.3%)	8 (17.8%)	0	31 (15.7%)
Type of diabetes					
Type 1	48 (50%)	0	4 (8.9%)	2 (4.5%)	54 (27.4%)
Type 2	43 (44.8%)	12 (100%)	39 (86.7%)	42 (95.4%)	136 (69%)
Unknown	5 (5.2%)	0	2 (4.4%)	0	7 (3.6%)
Transported by					
Self	22 (22.9%)	1 (8.3%)	8 (17.7%)	12 (27.3%)	43 (21.8%)
Ambulance	58 (60.4%)	9 (75%)	33 (73.3%)	26 (59%)	126 (63.9%)
Unknown	16 (16.7%)	2 (16.7%)	4 (8.9%)	6 (13.6%)	28 (14.3%)
Transported from					
Home	44 (45.8%)	4 (33.3%)	20 (44.4%)	37 (84%)	105 (53.3%)
Other health facility	39 (40.6%)	7 (58.3%)	21 (46.7%)	2 (4.5%)	69 (35%)
Unknown	13 (13.5%)	1 (8.3%)	4 (8.9%)	5 (11.4%)	23 (11.6%)
Presenting time					
Office hours^b^	32 (33.3%)	4 (33.3%)	18 (33.3%)	8 (18.2%)	62 (31.5%)
After hours	64 (66.7%)	8 (75%)	27 (60%)	36 (81.8%)	135 (68.5%)
Participant mobility					
Walking	33 (34.4%)	2 (16.7)	18 (40%)	3 (6.8%)	56 (28.4%)
With help	46 (47.9%)	4 (33.3%)	21 (46.7%)	13 (40.9%)	84 (42.6%)
Stretcher/immobile	14 (14.5%)	6 (50%)	6 (13.3%)	28 (63.6%)	54 (27.4%)
Unknown	3 (3.1%)	0	0	0	3 (1.6%)
Participant acuity^c^					
Non-urgent (green)	5 (5.2%)	0	2 (4.4%)	0	7 (3.5%)
Urgent (yellow)	20 (20.8%)	1 (8.3%)	0	9 (20.5%)	30 (15.2%)
Very urgent (orange)	39 (40.6%)	8 (75%)	2 (4.4%)	14 (31.8%)	63 (32%)
Emergent (red)	8 (8.3%)	2 (16.7%)	0	15 (34%)	25 (12.7%)
Unknown	24 (25%)	1 (8.3%)	41 (91.1%)	6 (13.6%)	72 (36.5%)

DKA, diabetic ketoacidosis; HHS, hyperosmolar hyperglycaemic state.

a 25th percentile to 75th percentile.

b Monday to Friday (08h00–15h59).

c According to Triage Early Warning Score (TEWS) of the South African Triage Scale (SATS).

Gastrointestinal symptoms, which includes at least one of either nausea, vomiting, abdominal pain and/or diarrhoea, occurred in most patients (n = 143, 72.6%). Infection and poor drug compliance were the most frequent precipitants (79 (40.1%) and 52 (26.4%) respectively). Most infections related to the respiratory tract (n = 23; 29.1%), and the gastro-intestinal system (n = 15; 19%). Other infections included soft tissue infections (n = 12; 16%), urinary tract infections (n = 11; 15%), undefined sepsis (n = 8; 10%), central nervous system infections (n = 5; 6.3%) and gynaecological infections (n = 3; 4%). In hypoglycaemic only participants, infection (n = 15; 34%), overmedication with prescribed hypoglycaemic agents (sulphonylureas and insulin) (n = 11, 25%), inadequate food intake (n = 9; 20.5%) and acute renal failure (n = 7, 15.9%) were the main reasons for presentation (Table 2).

**Table 2:** Precipitants, presenting symptoms and comorbidities in patients admitted with a diabetic emergency to the resuscitation area of Khayelitsha Hospital.

	DKA (n = 96) n (%)	HHS (n = 12) n (%)	Uncomplicated hyperglycaemia (n = 45) n (%)	Severe hypoglycaemia (n = 44) n (%)	Overall (n = 197) n (%)
Precipitant identified	86 (89.6%)	10 (83.3%)	37 (82.2%)	43 (97.7%)	175 (88.8%)
Precipitant					
Infection	36 (37.5%)	7 (58.3%)	21 (46.7%)	15 (34%)	79 (40.1%)
Non-compliance	38 (39.6%)	2 (16.7%)	12 (26.7%)	0	52 (26.4%)
Medication related	2 (2%)	0	0	11 (25%)	13 (6.6%)
Alcohol related	7 (7.3%)	0	0	1 (2.3%)	8 (4%)
Diet related	2 (2%)	0	0	9 (20.5%)	11 (5.6%)
Other	1 (1%)	1 (8.3%)	4 (8.9%)	7 (15.9%)	13 (6.6%)
Presenting signs and symptoms					
Gastrointestinal symptoms	95 (99%)	5 (41.7%)	33 (73.3%)	10 (22.7%)	143 (72.6%)
Polyuria & polydipsia	17 (17.7%)	1 (8.3%)	7 (15.5%)	0	22 (11.2%)
Lethargy	2 (2%)	0	0	2 (4.5%)	4 (2%)
Shortness of breath	19 (19.8%)	0	4 (8.9%)	4 (9%)	27 (13.7%)
Weakness	32 (33.3%)	6 (50%)	18 (40%)	4 (9%)	57 (28.9)
Altered level of consciousness	14 (14.6%)	10 (83.3%)	3 (6.7%)	26 (59%)	53 (26.9%)
Comorbidities					
Hypertension	31 (32%)	10 (83.3%)	28 (62.2%)	34 (77.3%)	103 (52.3%)
Chronic kidney disease	3 (3.1%)	2 (16.7%)	2 (4.4%)	7 (15.9%)	14 (7.1%)
HIV positive	19 (19.8%)	0	6 (13.3%)	7 (15.9%)	32 (16.2%)
Alcohol abuse/binge	11 (11.4%)	0	0	1 (2.3%)	12 (6%)
Macrovascular complications^a^	5 (5.2%)	3 (25%)	10 (22.2%)	10 (22.7%)	28 (12.7%)
Microvascular complications^b^	20 (20.8%)	7 (58.3%)	9 (20%)	19 (43.1%)	55 (27.9%)

DKA, diabetic ketoacidosis; HHS, hyperosmolar hyperglycaemic state; HIV, human immunodeficiency virus.

a Includes coronary artery disease, peripheral arterial disease, and stroke.

b Includes diabetic nephropathy, neuropathy, and retinopathy.

The investigations performed and treatment given are reported in Table 3. In total, 60 serum ketone tests were done of which 38 (63%%) were found to be positive. In the DKA group, 41 patients had a serum ketone test with urinary dipsticks used to diagnose 55 patients. Twenty (10.1%) of the 62 urine cultures sent were positive; the most common organisms were *Escherichia coli* (n = 5) and *Klebsiella pneumoniae* (n = 5). A total of 77 blood cultures were sent and 16 (21%) were positive; coagulase-negative staphylococcus was identified as the most common organism (n = 6), followed by *Klebsiella pneumoniae* (n = 2) and *Escherichia coli* (n= 2). The median (25th–75th percentile) amount of fluid given in all patients was 3.9 (2.0–5.6) L, and 72 (37%) patients received antibiotics (Table 3).

**Table 3:** Investigations done and treatment given for patients with a diabetic emergency managed in the resuscitation area of Khayelitsha Hospital.

	DKA (n = 96) n (%)	HHS (n = 12) n (%)	Uncomplicated hyperglycaemia (n = 45) n (%)	Severe hypoglycaemia (n = 44) n (%)	Overall (n = 197) n (%)
*Serum investigations*					
Blood gas	94 (97.9%)	12 (100%)	45 (100%)	35 (79.5%)	186 (94.4%)
Serum ketones done	40 (41.7%)	5 (41.7%)	13 (28.9%)	1 (2.3%)	59 (29.9%)
Positive serum ketones	29 (30.2%)	3 (25%)	7 (15.6%)	0	38 (17.8%)
Blood culture done	50 (52%)	10 (83.3%)	8 (17.8%)	9 (20.4%)	77 (39%)
Positive blood culture	9 (18%)	3 (30%)	0	4 (44.4%)	16 (20.8%)

*Other investigations*					
Urinary ketones	78 (81.3%)	8 (66.7%)	41 (91.1)	9 (20.4%)	136 (69%)
Positive urinary ketones	76 (97.4%)	2 (2%)	24 (58.5%)	1 (11.1%)	103 (75.7%)
Urine culture done	38 (39.6%)	7 (58.3%)	8 (17.7%)	9 (9%)	62 (31.4%)
Positive urine culture	11 (28.9%)	2 (28.6%)	3 (37.5%)	4 (44.4%)	20 (32.3%)
Sputum testing for pulmonary tuberculosis (Xpert MTB/RIF)	4 (4.2%)	0	3 (6.7%)	0	7 (3.6%)
Positive for pulmonary tuberculosis	2 (50%)	0	2 (66.7%)	0	4 (57.1%)
Electrocardiogram	73 (76%)	12 (100%)	21 (46.7%)	24 (54.4%)	130 (65.9%)
Chest radiograph (x-ray)	88 (91.7%)	12 (100%)	38 (84.4%)	39 (88.6%)	177 (89.3%)

*Treatment*					
Bolus insulin/sliding scale	16 (16.7%)	1 (8,3%)	36 (80%)	1 (2.3%)	54 (27.4%)
Insulin infusion	78 (81.3%)	11 (91,7%)	4 (8.9%)	1 (2.3%)	93 (47.2%)
Intravenous fluids	88 (91.7%)	12 (100%)	28 (62.2%)	28 (63.6%)	156 (79.2%)
Litres received (median (Q1-Q3))^a^	5.3 (3.2–7.2)	4.8 (2.55–6.35)	1.9 (1.0–2.25)	1.7 (1.0–2.0)	3.9 (2.0–5.6)
Antibiotics	39 (40.6%)	7 (58.3%)	13 (28.9%)	13 (29.5%)	72 (37%)

DKA, diabetic ketoacidosis; HHS, hyperosmolar hyperglycaemic state.

a 25th percentile to 75th percentile.

A summary of laboratory investigations are presented in Supplementary Table 1. Acute kidney injury occurred in 80 (40.6%) cases; 48 (60%) in the DKA group, 18 (23%) in the uncomplicated hyperglycaemia group, and eight (10%) in the HHS group. Six (13.6%) of the hypoglycaemic cases had acute kidney injury. Hyperkalaemia (potassium >5 mmol/L) occurred in 34 (22.2%) of all hyperglycaemic cases and hypokalaemia (potassium <3 mmol/L) in seven (4.6%) cases.

The median time spent in the resuscitation area was 8.4 h, with the longest times shared between patients with DKA and HHS (Table 4). Patients spent a median of 3.2 days in the hospital (Table 4). In-hospital teams managed 123 (62.4%) of the patients, with 20 (9.8%) of the patients transferred for tertiary care. The in-hospital mortality during the study period was 5.0% (n = 10; DKA n = 1; HHS n = 2; severe hypoglycaemia n = 7). Only one death occurred in the resuscitation area. While most deaths occurred in the hypoglycaemic group (n = 7, 70%), the actual cause of deaths were unrelated to diabetes and mainly related to an intracranial event (n = 3, 30%) or undefined sepsis (n = 4; 40%).

**Table 4:** Length of stay and disposition of patients managed with a diabetic emergency in the resuscitation area of Khayelitsha Hospital.

	DKA (n = 96) n (%)	HHS (n = 12) n (%)	Uncomplicated hyperglycaemia (n = 45) n (%)	Severe hypoglycaemia (n = 44) n (%)	Overall (n = 197) n (%)
Median length of stay (Q1-Q3)^a^					
Resuscitation area (h)	13 (7.2–24)	18 (7.2–24)	5 (2.4–9.6)	5 (2.4–7.2)	8.4 (3.8–18)
Hospital (days)	4.4 (2.1–7.6)	6.9 (5.3–8.9)	1.1 (0.5–4.0)	1.8 (0.8–5.4)	3.2 (0.9–6.3)

Disposition					
Discharged directly home	15 (13.5%)	0	24 (53.3%)	20 (45.4%)	57 (28.9%)
Referred to in-hospital disciplines	74 (77%)	10 (83.3%)	18 (40%)	21 (47.7%)	123 (62.4%)
Referred to tertiary facility	7 (7,3%)	2 (16.7%)	3 (16.7%)	2 (4.5%)	14 (7.1%)
Died in resuscitation area	0	0	0	1 (2.3%)	1 (0.5%)

DKA, diabetic ketoacidosis; HHS hyperosmolar hyperglycaemic state.

a 25th percentile to 75th percentile.

## Discussion

The prevalence of diabetic emergencies (8%) is indicative of the high burden on the resuscitation area of Khayelitsha Hospital. Almost half of the presentations related to DKA and urine dipsticks were mainly used to assess for ketosis. Infection was the main precipitant in all groups (40%). The high percentage of patients (51%) returning with a diabetic emergency to the emergency centre within six months is of concern and further increases the burden.

The prevalence of hyperglycaemic emergencies was 6.3% and 4.5% for DKA and HHS combined (i.e. uncomplicated hyperglycaemia excluded). This equates to about 18 patients per month, which is similar to a Canadian emergency centre where 17 patients with hyperglycaemic emergencies were treated [14]. However, the majority of patients had DKA (about 16 per month), which is substantially more than the ten patients per month with DKA seen in the emergency centre of a rural regional hospital in the KwaZulu/Natal province of South Africa [9]. Exact reasons for this difference remains unclear but could relate to geospatial differences (urban vs rural)in diabetes in general 15., 16..

More than 30% (60/197) of the participants returned within 30 days to the emergency centre with a diabetic- related emergency. This is higher than the 19% documented in Canada [14]. Recurrent hyperglycaemia visits have been associated with patients <25 years of age, a glucose level >20 mmol/L, being on insulin, and a recent visit to the emergency centre for hyperglycaemia [14]. Associated factors in our study still need to be formally explored, but it is clear that poorly controlled diabetes cause a great burden on the emergency centre of Khayelitsha Hospital as they typically require intensive monitoring, for potentially extended periods of time. Returning patients (especially those with DKA) are also at high risk of mortality, as seen in this study and prevention of recurrent presentations will thus benefit both the patient and the hospital [17].

Severe hypoglycaemic episodes were also regularly encountered in the resuscitation area (1.8 per 100 adults). This is similar to the 1.4 per 100 adult emergency centre visits in a US-based study [18]. Patients who experienced hypoglycaemic episodes were also likely to revisit the emergency centre; 50% returned within six months compared to 5% returning within 48 h in a US study [19]. Comparative data regarding hypoglycaemic episodes in South African emergency centres are lacking, but internationally patients using oral anti-diabetic agents are at higher risk to return to the emergency centre than those treated with insulin alone [19].

Urinary ketone assessment (using a dipstick) was more frequently used than testing for serum ketones (laboratory done) to detect the presence of ketosis in hyperglycaemic patients (urine 127/153, 83%; serum 58/153, 38%). The presence of ketones in the urine (ketonuria) is often used as a surrogate marker for serum ketones (ketonemia) [7], as serum ketone laboratory tests are more costly and not everywhere readily available. However, there are two ketones that play a role in DKA, acetoacetate and beta-hydroxybutyrate, with the latter being the predominant ketone in severe untreated DKA. Acetoacetate is detected in urinary dipsticks, while beta-hydroxybutyrate is not [20]. Point-of-care blood beta- hydroxybutyrate strips are available, although not at Khayelitsha Hospital at the time of the study. A US-based study found a similar sensitivity for blood beta-hydroxybutyrate strips and urine acetoacetate dipsticks (both 98%) in diagnosing DKA, but blood beta-hydroxybutyrate strips had higher specificity (79%) than the urine acetoacetate dipsticks (35%) [21]. The decreased specificity of the urine dipstick raises concerns of potentially unwarranted medical work-ups as a result of false negative tests.

Infection was deemed to be the precipitant factor in 40% of patients which confirms the high rate of infection in patients presenting with diabetic emergencies [22], 23., [24]. This further indicates the need for the diligent search for infection, which should include cultures of blood, urine and pulmonary specimens. One in every five blood cultures done were positive, and coagulase-negative staphylococcus was the most common organism isolated. However, this is often a contaminant and clinical correlation is needed to discern if a bacteraemia is being caused by this organism. The yield in urine cultures was slightly higher (1.5 in every five), and although only a few sputum cultures were collected, more than half (57.1%) was positive for tuberculosis. Diabetics are predisposed to infection and increased, and specialised screening programmes should be considered in settings with a high tuberculosis prevalence 25., 26..

In an attempt to lessen the burden of diabetic emergencies on hospitals, it is essential that an integrated approach be followed, which involves community based programmes. Currently not much is known about the effectivity and its adaptability of existing programmes available in this community. Community health care workers are often employed by non-governmental organisations in South Africa [27]. In 2011, there were 1.1 non-governmental organisations per 10,000 population in Khayelitsha; most not covering non-communicable diseases [27]. Furthermore, one study indicated that the knowledge of community health care workers in Khayelitsha regarding diabetes was poor, with only 52% of the community health care workers reporting that they received formal training related to non-communicable diseases. It is clear that much improvement is needed if community-based management of diabetes is to be successful [27], 28..

A strength of the study was the use of a pre-piloted standardised data collection form. However, the results could have been affected by various limitations. Firstly, the study reflects the experience at a single urban emergency centre with standardised protocols and care should be taken to generalise the results to different settings. The retrospective nature of the study led to the reliance on adequate documentation for quality data. Participants could have received treatment at referring clinics or from ambulance crews before arrival; this could have led to specific parameters being different by the time the patients presented to the hospital. Included weeks were randomly selected from the existing database, and seasonal variation could have influenced the prevalence. Lastly, definitions from the American Diabetes Association (ADA) and the Society for Endocrinology, Metabolism and Diabetes of South Africa (SEMDSA) were used and did not include euglycaemic DKA as part of its criteria; this could allude to an underrepresentation of DKA patients.

This study highlights the high burden of diabetic emergencies on the provision of acute care at a district-level hospital. The high prevalence of diabetic emergencies (8%) consisted of DKA (48.7%), uncomplicated hyperglycaemia (22.8%), severe hypoglycaemia (22.3%), and HHS (6%). The high infection rate (40%) and the high percentage of patients returning with a diabetic emergency (51%) could be indicative of the need for improved community-based diabetic programmes.

The following is the supplementary data related to this article.

Supplementary Table 1 Summary of laboratory investigations done at presentation in patients with a diabetic emergency managed in the resuscitation area of Khayelitsha Hospital. Supplementary Table 1

## Dissemination of results

Results from this study were shared with staff members at the data collection site through an informal presentation.

## Authorship contribution statement

Authors contributed as follow to the conception or design of the work; the acquisition, analysis, or interpretation of data for the work; and drafting the work or revising it critically for important intellectual content: NL contributed 70%; SL 15%; and DJvH contributed 15%. All authors approved the version to be published and agreed to be accountable for all aspects of the work.

## Declaration of competing interest

The authors declared no conflicts of interest.
