# Supplementary material for: The burden of diabetic emergencies on the resuscitation area of a district-level public hospital in Cape Town
Source: Afr J Emerg Med. 2021 Oct 14;11(4):416–21. doi: 10.1016/j.afjem.2021.05.004 (PMC8524109; doi:10.1016/j.afjem.2021.05.004)
Supplement: Supplementary Table 1 — Summary of laboratory investigations done at presentation in patients with a diabetic emergency managed in the resuscitation area of Khayelitsha Hospital. [file mmc1.docx]

**Supplementary material**

**Supplementary Table 1** Summary of laboratory investigations done at presentation in patients with a diabetic emergency managed in the resuscitation area of Khayelitsha Hospital

| **Mean±SD unless otherwise stated** | **Normal range** | **DKA (n=96)**  **n (%)** | **HHS**  **(n=12)**  **n(%)** | **Uncomplicated hyperglycaemia (n=45)**  **n (%)** | **Severe hypoglycaemia (n=44)**  **n (%)** | **Overall (n=197)**  **n (%)** |
| --- | --- | --- | --- | --- | --- | --- |
| pH | 7.35-7.45 | 7.1±0.18 | 7.2±0.15 | 7.38±0.06 | 7.3±0.1 | 7.2±0.18 |
| Bicarbonate  (mmol/L) | 18-28 | 12.7±8.6 | 16.6±6.3 | 23.7±3 | 21.1±7.9 | 16.4 ±8.9 |
| Base excess (meq/l) | -2 to +2 | -12.2±11.2 | -9.1±8 | -0.12±3.8 | -3±9.1 | -7.3±10.7 |
| Lactate (mmol/L) | <2 | 3.2±1.9 | 4.1±3.6 | 1.98±0.8 | 2.7±3.2 | 3±2.4 |
| Haematocrit (%) | 36-46 | 47.3±8.8 | 45.5±8.4 | 40.8±7.7 | 36.1±7.9 | 43.6±9.4 |
| Sodium (mmol/L) (median((Q1-Q3))d | 135-145 | 130(126-134.5) | 127.5(118-136.3) | 130.5  (127-134.8) | 135  (131.5-137.5) | 131  (127-135) |
| Potassium (mmol/L)  (median ((Q1-Q3))d | 3.5-5.5 | 5.0(4.4-5.9) | 5.3(4.6-6.6) | 4.4(4.8-4.8) | 4.4(3.9-5.2) | 4.7(4.2-5.7) |
| Urea (mmol/L) (median ((Q1- Q3))d | 2.5-7.1 | 7.10(5.5-11.7) | 26.1  (17.2-36.6) | 7.0  (4.0-4.8) | 9.0  (5.1-20.0) | 7.9  (5-15.9) |
| Creatinine (mmol/L) (median ((Q1-Q3))d | 60-120 | 112  (90.5-168) | 310  (188.5-  448) | 95  (71-128) | 120.50  (60.5-212.3) | 113.5  (77.8-179.3) |
| White cell count (x 109/L) | 3.9-  12.6 | 19±12 | 15.4±8.1 | 10.5±5 | 8.7±4 | 14.7±10.3 |
| Haemoglobin (g/dL) (median (Q1-Q3)* | 12.0-  15.0 | 13.8  (11.5-15.0) | 12.7  (10.3-14.9) | 12.3  (10.2-13.1) | 10.3  (9.2-11.5) | 12.5  (10.2-14.4) |
| Platelets (x 109/L) | 186-  454 | 368.4±167.7 | 271.7±76.9 | 334±139.8 | 329.5±116.1 | 345.8±148.4 |
| Haemoglobin A1c (%) | <7 | 13.4±5.6 | 11.6 ±4.9 | 12.9±3.2 | 8.3±2.4 | 12.1±5.1 |

SD, standard deviation; DKA, Diabetic Ketoacidosis; HHS, Hyperosmolar hyperglycaemic state; *, 25th percentile to 75th percentile
